# Phylodynamic of Tomato Brown Rugose Fruit Virus and Tomato Chlorosis Virus, Two Emergent Viruses in Mixed Infections in Argentina

**DOI:** 10.3390/v17040533

**Published:** 2025-04-05

**Authors:** Julia M. Ibañez, Romina Zambrana, Pamela Carreras, Verónica Obregón, José M. Irazoqui, Pablo A. Vera, Tatiana E. Lattar, María D. Blanco Fernández, Andrea F. Puebla, Ariel F. Amadio, Carolina Torres, Paola M. López Lambertini

**Affiliations:** 1 Estación Experimental Agropecuaria Bella Vista, Instituto Nacional de Tecnología Agropecuaria (INTA), Ruta 27-Km 38,3, Bella Vista, Corrientes 3432, Argentina; ibanez.magali@inta.gob.ar (J.M.I.); obregon.veronica@inta.gob.ar (V.O.); lattar.tatiana@inta.gob.ar (T.E.L.); 2 Instituto de Investigaciones en Bacteriología y Virología Molecular (IBaViM), Facultad de Farmacia y Bioquímica, Universidad de Buenos Aires, Junin 956, 4th floor, Ciudad Autónoma de Buenos Aires 1113, Argentina; rzambrana@docente.ffyb.uba.ar (R.Z.); dolobf@gmail.com (M.D.B.F.); caro.torr@gmail.com (C.T.); 3 Instituto de Patología Vegetal, Centro de Investigaciones Agropecuarias, Instituto Nacional de Tecnología Agropecuaria (IPAVE-CIAP-INTA), Av. 11 de Septiembre, X5014MGO, Córdoba 4755, Argentina; carreras.pamela@inta.gob.ar; 4 Unidad de Fitopatología y Modelización Agrícola (UFYMA) INTA-Consejo Nacional de Investigaciones Científicas y Técnicas (CONICET), Av. 11 de Septiembre, X5014MGO, Córdoba 4755, Argentina; 5 Instituto de Investigaciones de la Cadena Láctea (IDICAL) INTA-CONICET, Ruta 34 km 227, Rafaela, Santa Fe 2300, Argentina; irazoqui.jose@inta.gob.ar (J.M.I.); amadio.ariel@inta.gob.ar (A.F.A.); 6 Unidad de Genómica y Bioinformática (UGB), Instituto de Agrobiotecnología y Biología Molecular (IABiMo), INTA-CONICET, De los Reseros y N. Repetto, Hurlingham, Ciudad Autónoma de Buenos Aires 1686, Argentina; vera.pablo@inta.gob.ar (P.A.V.); puebla.andrea@inta.gob.ar (A.F.P.)

**Keywords:** ToBRFV, ToCV, tomato, wastewater viromics, emerging viruses, whole-genome sequencing, viral phylodynamics, Bayesian coalescent analysis, plant viral outbreaks, virus evolution

## Abstract

*Tobamovirus fructirugosum* (ToBRFV) and *Crinivirus tomatichlorosis* (ToCV) are emerging viral threats to tomato production worldwide, with expanding global distribution. Both viruses exhibit distinct biological characteristics and transmission mechanisms that influence their spread. This study aimed to reconstruct the complete genomes of ToBRFV and ToCV from infected tomato plants and wastewater samples in Argentina to explore their global evolutionary dynamics. Additionally, it compared the genetic diversity of ToBRFV in plant tissue and sewage samples. Using metagenomic analysis, the complete genome sequences of two ToBRFV isolates and two ToCV isolates from co-infected tomatoes, along with four ToBRFV isolates from sewage, were obtained. The analysis showed that ToBRFV exhibited higher genetic diversity in environmental samples than in plant samples. Phylodynamic analysis indicated that both viruses had a recent, single introduction in Argentina but predicted different times for ancestral diversification. The evolutionary analysis estimated that ToBRFV began its global diversification in June 2013 in Israel, with rapid diversification and exponential growth until 2020, after which the effective population size declined. Moreover, ToCV’s global expansion was characterized by exponential growth from 1979 to 2010, with Turkey identified as the most probable location with the current data available. This study highlights how sequencing and monitoring plant viruses can enhance our understanding of their global spread and impact on agriculture.

## 1. Introduction

*Tobamovirus fructirugosum* (tomato brown rugose fruit virus, ToBRFV) and *Crinivirus tomatichlorosis* (tomato chlorosis virus, ToCV) are emerging viral pathogens in Argentina, with their global distribution expanding rapidly. Both viruses significantly impact tomato yields worldwide, particularly in Argentina, where the national tomato production in 2022 was reported at 71,638 kg/ha across 19,445 hectares (FAO, 2022: https://www.fao.org/faostat/en/#data/QCL, accessed on 27 June 2024). ToBRFV was first identified in Israel and Jordan between 2014 and 2015 and has since spread to four continents [1,2]. In Argentina, ToBRFV was first identified in Corrientes in 2022, where it was found infecting greenhouse-grown tomatoes [3]. ToBRFV belongs to the genus *Tobamovirus* and possesses a genome composed of a single-stranded, positive-sense RNA with approximately 6400 nucleotides (nt) in length, encapsidated in a rod-shaped particle. The genome contains four open reading frames (ORFs), each encoding proteins involved in replication, movement, encapsidation, and the suppression of host gene silencing mechanism [4]. ToBRFV is seed-borne and mechanically transmissible [4]. Additionally, the viral particles demonstrate stability, thereby enhancing the potential for their transmission across crops and enabling their prolonged persistence on a variety of surfaces, such as greenhouse infrastructure, tools, clothing, soil, and water [5,6,7]. Although the ToBRFV seed transmission rate is relatively low (ranging from 0.08% to 2.8%), it can serve as a significant primary source of inoculum, driving its global spread [8,9]. ToBRFV induces symptoms such as mosaic patterns, mottling, and leaf narrowing. Necrosis may also occur on the stems. However, the most severe symptoms manifest in the tomato fruit, where brown rugosity, wrinkled patches, discoloration, and deformation are observed [4]. To date, ToBRFV has been reported in natural infections in tomato (*Solanum lycopersicum*), pepper (*Capsicum annuum*), and the weeds *Solanum nigrum*, *Convolvulus arvensis*, and *Polycarpon tetraphyllum* [10,11]. Additionally, numerous alternative weed hosts have been suggested based on prior artificial experimental studies involving mechanical inoculation [12,13]. There have been reports suggesting that the tomato moth (*Tuta absoluta*) and the bumblebee (*Bombus terrestris*) may facilitate the spread of ToBRFV; however, they do not play a vector role [14,15].

ToCV was first described in Florida (USA) in 1996, where it was found infecting tomatoes, and has since spread globally [16,17]. ToCV is a member of the *Crinivirus* genus, and its genome consists of two single-stranded, positive-sense RNA molecules (RNA1 and RNA2), each encapsidated separately in long, flexuous, rod-like particles [18]. RNA1 is approximately 8596 nucleotides in length and contains four ORFs that encode proteins involved in virus replication and the suppression of gene silencing [19]. RNA2, with a length of around 8247 nucleotides, contains nine ORFs that encode proteins potentially associated with virus encapsidation, whitefly transmission, membrane interaction, cell-to-cell movement, and the suppression of gene silencing [19]. ToCV is not transmitted through seeds or mechanically; instead, it is transmitted in a semi-persistent manner by four whitefly species belonging to two genera (*Bemisia* and *Trialeurodes*) [20]. *Bemisia tabaci* MED (Mediterranean) exhibits a higher virus acquisition and accumulation rate, as well as superior performance (e.g., fecundity), resulting in the more efficient transmission of ToCV compared to *Trialeurodes abutilonea* and *B. tabaci* MEAM1 (Middle East–Asia Minor 1). The less efficient vectors include *B. tabaci* New World 1 and *Trialeurodes vaporariorum* [20,21,22]. Symptoms caused by ToCV in tomato plants initially manifest on the lower leaves, which display interveinal chlorosis, alternating green and yellow patches. As the disease progresses to the upper parts of the plant, yellow areas with reddish-brown necrotic flecks appear. The lower leaves typically become rolled, thickened, and crispy to the touch. While no overt symptoms are visible on the fruit, yield loss occurs due to reduced fruit growth and delayed ripening [18]. Due to its broad host range, which includes 119 plant species across 28 botanical families, ToCV affects several economically important crops such as sweet pepper, eggplant, potato, tobacco, pumpkin, lettuce, and others, as well as numerous common, widely distributed weeds [17].

Visualizing viral genomic data derived from plant materials and environmental sources in relation to geographic distribution provides a robust tool for advancing our comprehension of pathogen dynamics. Indeed, several studies have highlighted the predictive potential of wastewater surveillance for plant viruses, demonstrating its capacity to monitor and prevent plant viral disease outbreaks, particularly for stable viral particles such as tobamoviruses [23,24,25]. Other studies have also reported the detection of ToBRFV RNA in wastewater samples, as well as in river water and water used for irrigation [6,25,26]. However, to date, no studies have investigated the origin and dispersion of ToBRFV and ToCV using phylodynamic integrative approaches that incorporate ancestral dating estimation, demographic reconstruction, and phylogeographic analysis.

This study sought to reconstruct the complete genomes of ToBRFV and ToCV from infected tomato plants and wastewater samples in Argentina, with the objective of elucidating the global evolutionary dynamics of these viruses. Additionally, it aimed to compare the intra-sample genetic diversity of ToBRFV in plant tissue and raw sewage samples.

## 2. Materials and Methods

### 2.1. Plant Collection and High-Throughput Sequencing

**Figure 1:**
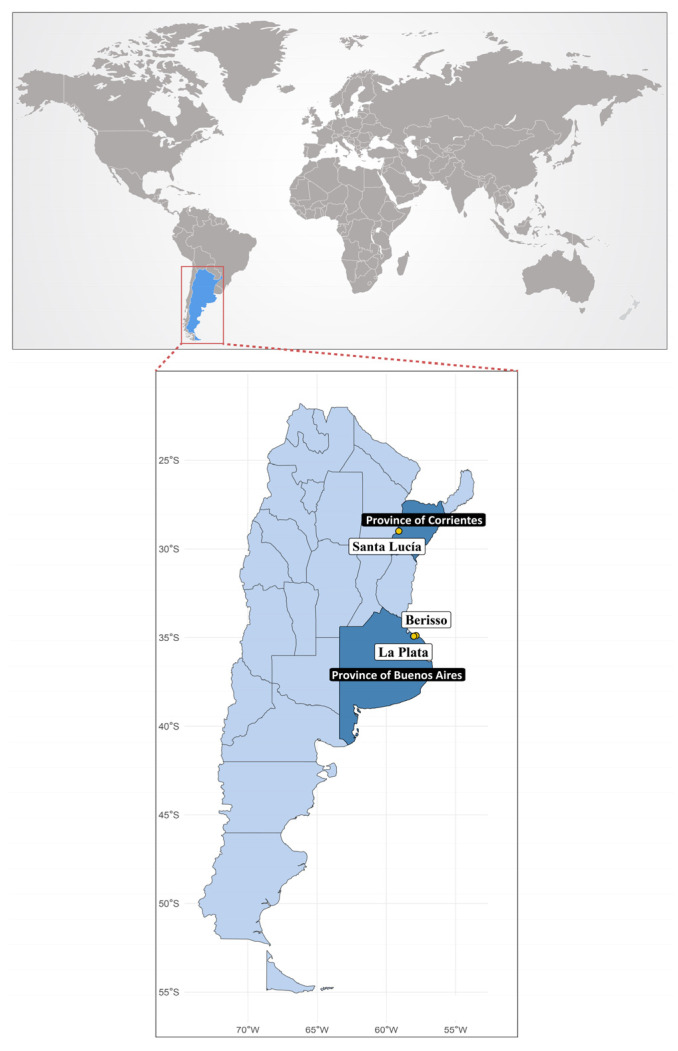
Map of Argentina sampling sites. Yellow dots indicate sampling locations and white labels show the names of sampling cities located within Argentine provinces, colored in dark blue.

Symptomatic tomato plants cultivated under greenhouse conditions were sampled from two prominent fresh tomato production areas in Argentina, the city of Santa Lucía (Province of Corrientes) and the city of La Plata (Province of Buenos Aires), during 2023 (Figure 1). Initial screening was performed using ImmunoStrip tests (Agdia Inc., Elkhart, IN, USA) for ToBRFV on five and seven samples, respectively. Based on symptom presentation and the preliminary results from the ImmunoStrip assays, one sample from each location was selected for high-throughput sequencing (HTS). Total RNA was extracted from 100 mg of ground leaf tissue for each sample using TRIzol Reagent (ThermoFisher, Whaltam, MA, USA), followed by ribosomal RNA depletion using a Ribo-Zero Plant rRNA removal kit (Illumina, San Diego, CA, USA). NGS libraries were constructed using a TruSeq RNA Library Prep Kit (Illumina) according to the manufacturer’s instructions. The libraries were sequenced on the NovaSeq 6000 platform (Genomic and Bioinformatics Unit of ANLIS), generating 50 million paired-end 2 × 150 reads per library.

### 2.2. ToBRFV and ToCV Complete Genome Reconstruction from Leaves Samples

Reads were quality-trimmed, and sequencing adapters were removed using TrimGalore (https://github.com/FelixKrueger/TrimGalore, accessed on 15 April 2024). Transcripts abundance was first estimated using Trinity and Salmon [27,28]. The most abundant transcripts were then compared against the non-redundant (nr) database using BLAST es 2.15.0 to quantify viral transcript presence. To obtain the complete viral genomes, reads were mapped to the tomato reference genome (GCF_000188115.5) using Bowtie2, with results processed using Samtools [29,30]. Unmapped reads were assembled *de novo* using rnaSPAdes, and the resulting contigs were compared against the nr database using BLAST [31]. After viral species identification, open reading frames (ORFs) across all genomes were examined to evaluate the assembly quality. To further refine the data, reads were mapped against the assembled contigs using Bowtie2, and regions with coverage below 50 were removed to eliminate low-confidence regions.

### 2.3. Environmental Water Sample Collection, Processing, and Viral RNA Extraction

Four raw sewage samples (2 L each) were collected from the influent channel of a Wastewater Treatment Plant (WWTP) in the City of Berisso, Province of Buenos Aires, Argentina, during February, March, October, and December of 2022 (Figure 1). This WWTP serves a population of at least 200,000 people from Berisso and the surrounding areas, including La Plata, a major agricultural production region. Notably, the WWTP’s location, in the province of Buenos Aires, is near one of the locations where tomatoes were sampled. These wastewater samples were collected as part of a separate virome study conducted in Argentina and were reanalyzed within the framework of this study. The samples were promptly transported to the laboratory, where a virus concentration protocol was implemented as described by ref. [32]. Briefly, each sample was pre-filtered using 0.45 μm and 0.22 μm filters, followed by ultrafiltration with a 50 kDa membrane. Polyethylene glycol (PEG) 6000 was added to a final concentration of 10%, along with 3% NaCl. The mixture was incubated overnight at 4 °C, and after centrifugation (15 min, 10,000× *g*, 4 °C), the resulting pellet was resuspended in 2 mL PBS and stored at −80 °C. To remove unprotected free DNA, 300 μL of the sample was treated with DNase I (final concentration of 10 U/mL, New England Biolabs, Ipswich, MA, USA) for 10 min at 37 °C. Viral RNA was extracted using the QIAamp Viral RNA Mini Kit (QIAGEN, Germantown, MD, USA) according to the manufacturer’s instructions and eluted in 60 μL of AVE buffer. RNA extracts were used as input for cDNA synthesis and subsequent PCR amplification via a sequence-independent single-primer amplification (SISPA) approach, involving two rounds of PCR [33]. For reverse transcription, 2 μL of Primer A (50 μM) (5′-GTTTCCCAGTCACGATC-N9-3′) was used with SuperScript III Reverse Transcriptase (Thermo Fisher Scientific Inc. Whaltam, MA, USA). Following second-strand cDNA synthesis using Klenow polymerase (New England Biolabs, Ipswich, MA, USA), the products were purified with the Wizard SV Gel and PCR Clean-Up System Kit (Promega Corp, Madison, WI, USA), following the manufacturer’s instructions. The purified DNA products were then amplified using Q5 High-Fidelity DNA Polymerase (New England Biolabs, Ipswich, MA, USA) in two successive rounds of PCR. In the first round, Primer A was used under the following conditions: 94 °C for 3 min, followed by 30 amplification cycles (94 °C for 30 s, 37 °C for 30 s, and 72 °C for 2 min), and a final elongation cycle at 72 °C for 5 min. In the second round of PCR, Primer B (5′-GTTTCCCAGTCACGATC-3′) was used, with the following conditions: 94 °C for 3 min, 35 amplification cycles (94 °C for 30 s, 54 °C for 30 s, and 72 °C for 2 min), and a final extension at 72 °C for 5 min. PCR products were purified using the Wizard SV Gel and PCR Clean-Up System Kit (Promega Corp, Madison, WI, USA).

### 2.4. ToBRFV Genome Reconstruction from Raw Sewage Samples

Samples were paired-end sequenced on the Illumina platform (read length 150 bp) at Novogene Bioinformatics Technology Co., Ltd. Raw reads were processed to remove adapters and primers A/B using TrimGalore on the Galaxy Europe platform [34]. Additionally, short reads (<75 bp) and low-quality nucleotides (Phred score below 30) were discarded. Taxonomic classification and organism abundance estimation of clean sequences were conducted using Kraken2 and Bracken, respectively, against the NCBI RefSeq Viral database on the Galaxy Europe platform [35,36]. Following the detection of ToBRFV, clean reads were aligned to the ToBRFV RefSeq complete genome (NC_028478.1) using BWA v.0.7.17 [37]. The consensus genome for each sample was constructed by selecting the most frequent nucleotide per position, applying a minimum quality score threshold of 20, using Samtools v.1.13 and iVar v1.4.2 software [38,39].

### 2.5. Phylodynamic Analysis of ToBRFV

Ancestral locations, divergence times (time to the most recent common ancestors, tMRCAs), and the evolutionary rate of ToBRFV were estimated using Bayesian inference, implemented in BEAST v1.10.4 on the CIPRES Science Gateway [40,41].

Initially, 230 complete genomes were retrieved from the NCBI Virus Database (https://www.ncbi.nlm.nih.gov/labs/virus/vssi/#/, accessed by 24 April 2024). Considering geographic representation and prioritizing genomes with collection location and date information, a subsampling was performed, resulting in a dataset of 164 genomes. Then, Argentine ToBRFV consensus sequences obtained from leaf samples (*n* = 2) and sewage samples (*n* = 4) were included. Sequences were aligned using MAFFT v.7.526, and the alignment was manually curated to remove the 5′ and 3′ untranslated regions (UTRs) [42]. The best-fitting nucleotide substitution model was selected using ModelFinder in the IQ-TREE v2.3.2 software [43,44]. The temporal structure of the refined dataset (*n* = 170) was confirmed with root-to-tip regression analysis using TempEst v1.5.3 [45]. Phylodynamic analysis, incorporating phylogeography, was performed using BEAST v1.10.4 with an uncorrelated log-normal (UCLN) relaxed clock and the GMRF Bayesian Skyride coalescent model [46,47]. A spatial diffusion process was modeled across time-measured genealogies, using discrete sampling locations (countries of origin of samples) and an asymmetric diffusion model [48]. Two independent MCMC chains were run, each of 200 million generations with sampling every 200,000 steps. After discarding the first 10% of samples as burn-in, MCMC chain convergence was assessed by confirming an effective sample size (ESS) greater than 200 using Tracer 1.7 [49]. The results from both runs were then combined, and the annotated Maximum Clade Credibility (MCC) tree was generated using Tree Annotator v1.10.4 and visualized with FigTree v1.4.4 (available at: http://tree.bio.ed.ac.uk/software/figtree/, accessed on 1 May 2024).

### 2.6. Phylogenetic and Phylodynamic Analysis of ToCV

Phylogenetic trees of ToCV were constructed using the coding sequences of RNA1 (*n* = 48) and RNA2 (*n* = 48). Each dataset included ToCV sequences downloaded from the NCBI Virus Database (https://www.ncbi.nlm.nih.gov/labs/virus/vssi/#/, accessed on 17 May 2024), as well as Argentine ToCV sequences obtained from leaf isolates (*n* = 2). Multiple sequence alignments were performed using MAFFT v.7.526 with default parameters and phylogenetic trees were generated using IQ-TREE v2.3.2 software [43,44]. Tree confidence was assessed using Ultrafast Bootstrap approximation (UFBoot) and the Shimodaira–Hasegawa approximate likelihood ratio test (SH-aLRT) with 10,000 and 1000 replicates, respectively [50,51].

To investigate the evolutionary dynamics of ToCV, the temporal structure of RNA1 and RNA2 datasets was explored using TempEst v1.5.3, although no positive relationship between divergence and time was observed in the root-to-tip regression analysis, precluding the calibration of the phylodynamic analysis using sampling times. Therefore, a new dataset of coat protein (CP) sequences was compiled, consisting of 153 ToCV CP sequences from the NCBI Virus Database (https://www.ncbi.nlm.nih.gov/labs/virus/vssi/#/, accessed by 16 July 2024), along with two Argentine ToCV sequences obtained from leaf isolates. The temporal structure of this dataset (*n* = 155) was confirmed through a root-to-tip regression analysis using TempEst v1.5.3, and phylodynamic analyses were performed as previously outlined.

### 2.7. Intra-Sample Diversity in Argentine ToBRFV Sequences

The viral intra-sample variation in Argentine ToBRFV sequences was studied by estimating the intra-sample single nucleotide variants (iSNV) from the aligned reads against the ToBRFV reference genome (NCBI RefSeq Accession Number: NC_028478.1) using the iVar software [39]. The iSNVs were identified based on the following criteria: variant frequency higher than 1% (option -t 0.01), at positions with a minimum depth of 10 reads (option -m 10), and with *p*-values for Fisher’s exact test higher than 0.01 (option PASS = TRUE, used to determine if the iSNV frequency is significantly higher than the mean error in the position, according to its base Q and depth). To analyze minority frequency variants, only the iSNVs with frequencies between 3% and 50% were considered.

## 3. Results

### 3.1. Viral Complete Genome Sequences Through Metagenomics Analysis

**Figure 2:**
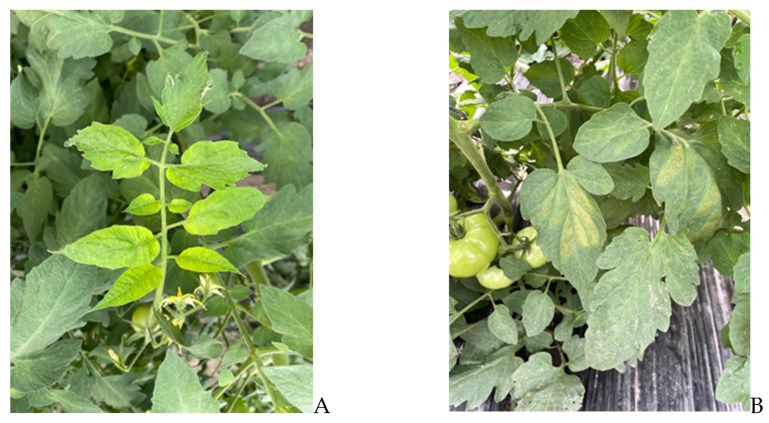
(**A**) Symptoms of chlorosis on tomato leaves infected with ToBRFV and ToCV, as observed in samples from Province of Buenos Aires. (**B**) Symptoms of irregular chlorotic and interveinal spots and small dark lesions on tomato leaves infected with ToBRFV and ToCV, as observed in samples from Province of Corrientes.

The symptoms observed in tomato plants sampled in the Province of Buenos Aires were characterized by irregular chlorotic patterns and interveinal spots across both halves of the leaflet. These symptoms initially appear in the lower and middle sections of the plant (Figure 2A). Fruit symptoms included uneven ripening and green blotchy areas on mature fruits. In contrast, the symptoms in tomato plants sampled from Province of Corrientes involve a more widespread yellowing as the crop matures, with the veins remaining dark green. Occasionally, small dark lesions with a faint violet tint form in the chlorotic areas (Figure 2B). The fruits exhibit necrotic lesions, which can develop in both green immature fruits and mature fruits.

The full-length sequences of two ToBRFV isolates and two ToCV isolates infecting tomatoes were obtained using metagenomics analysis. ToBRFV and ToCV were detected in mixed infections in tomatoes collected from the Provinces of Corrientes and Buenos Aires, two prominent tomato production regions in Argentina (Figure 1). Additionally, four complete ToBRFV genomes, represented as consensus sequences, were obtained from wastewater samples collected in four distinct months from the influent channel of a Wastewater Treatment Plant in the City of Berisso, near the City of La Plata in the Province of Buenos Aires (Figure 1). However, no sequences corresponding to ToCV were detected in these samples.

### 3.2. Spatial and Temporal Dynamics of ToBRFV

**Figure 3:**
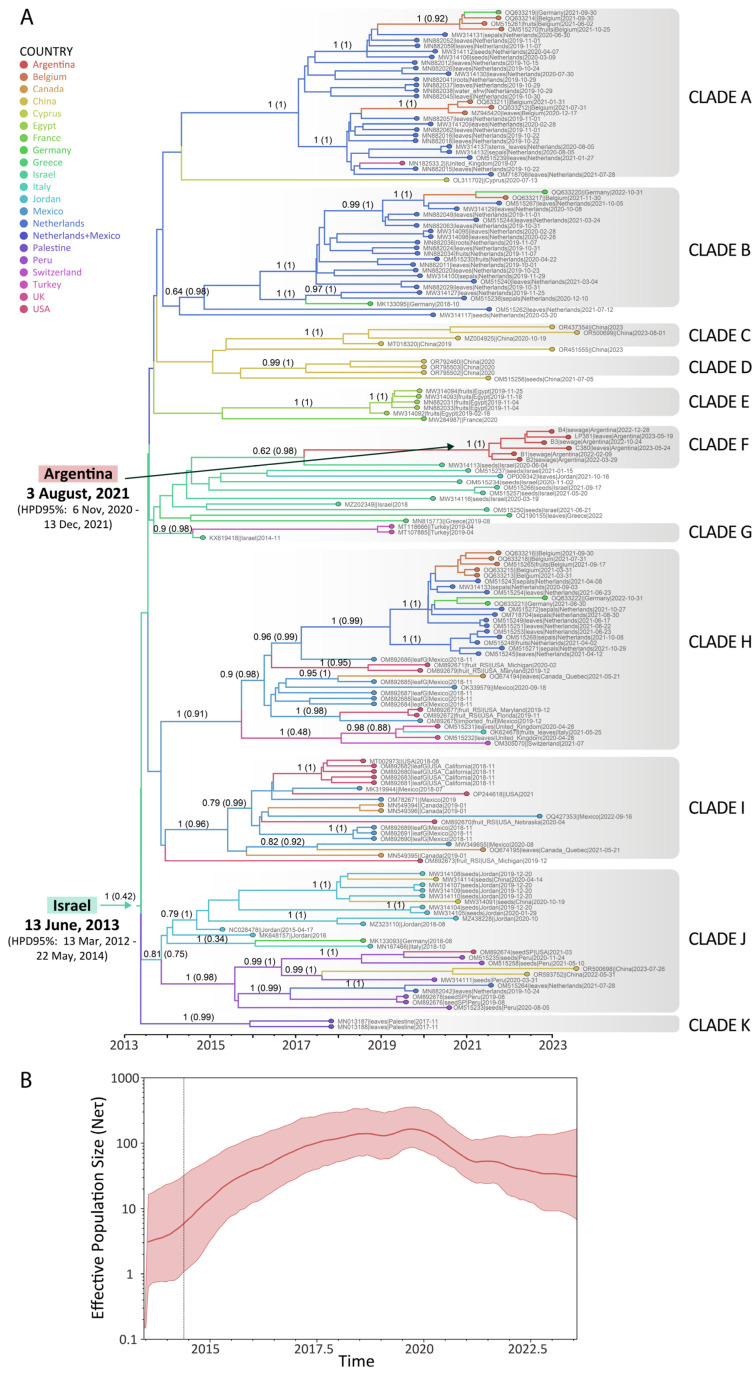
Phylodynamic analysis of ToBRFV. (**A**) Maximum clade credibility tree for ToBRFV complete genome sequences. Posterior probability values > 0.6 are shown on the branches along with the posterior probability of the ancestral location (ppal) in brackets. The time scale is in years. (**B**) Demographic reconstruction of ToBRFV complete genome sequences. The violet line shows the median value, and the colored areas represent the 95%HPD interval of the estimated effective population size. The *x*-axis is the time in years, and the *y*-axis is on a log scale (effective population size (Ne) × generation time (Ƭ)).

As illustrated in Figure 3A, which depicts the dated tree of ToBRFV, the clades were identified based on the grouping and support provided by the Bayesian analysis (posterior probabilities). ToBRFV sequences tend to group according to their geographical region but not exclusively. It can be distinguished in countries with several introductions, like the Netherlands and Germany (Clade A, B, H and J), Belgium (Clade A, B and H), China (Clade C, D, and J), the United Kingdom (Clade A and H), the USA (Clade H, I and J), Mexico (Clade H and I), Canada (Clade H and I), Jordan (Clade J and a sequence outside a clade), and Italy (Clade H and J), as well as in countries in which it one introduction was detected with further dispersion, such as Egypt (Clade E), Argentina (Clade F), Turkey (Clade G), Peru (Clade J), and Palestine (Clade K). In addition, some countries were represented only by one sequence, such as France (Clade E) and Switzerland (Clade H), or by sequences closely related but not grouped in a supported clade, such as Greece (two sequences related to Clades F and G) or Israel (one sequence in Clade F, one sequence in Clade G and others intermingled between them) (Figure 3A).

In the case of South American sequences, phylogeographic analysis placed them in two distinct clades: Clade F, grouping ToBRFV genomes from Argentina with genomes from Israel, and Clade J, grouping ToBRFV sequences from Peru, with sequences from Jordan, China, Germany, Italy, the Netherlands, and the USA (Figure 3A). Simultaneously, Clade F, which encompasses the six genome sequences reported in this study, suggests a single viral introduction into the country with further dispersion (Figure 3A). The Argentinean ToBRFV isolates share a common ancestor with ToBRFV isolated from *Capsicum annuum* seed collected in 2020 in Israel (MW314113), although this group showed moderate support (posterior probability = 0.62). In particular, the sequences from Argentina would have started their diversification in the country at the beginning of August 2021 (HPD95% = November 2020–December 2021), from a virus initially located in Israel (posterior probability of the ancestral location (ppal) = 0.98), and are related to viruses that circulated in that country between 2020 and 2021 (Figure 3A).

In addition, the analysis estimated that ToBRFV began its global diversification in June 2013 (HPD95% = March 2012–May 2014), with Israel as the most probable location, although with a low probability (ppal= 0.42), followed by Jordan (ppal = 0.28) (Figure 3A). Further, the rapid diversification and exponential growth of ToBRFV were observed until 2020, after which the effective population size declined slightly (Figure 3B). Notably, the outbreak of ToBRFV in Argentina occurred during this period of decline (Figure 3B). Furthermore, the overall substitution rate for ToBRFV was estimated at 2.9 × 10−4 substitutions per site per year (s/s/y) (95%HPD = 2.2 × 10−4 − 3.6 × 10^−4^).

### 3.3. Spatial and Temporal Dynamics of ToCV

**Figure 4:**
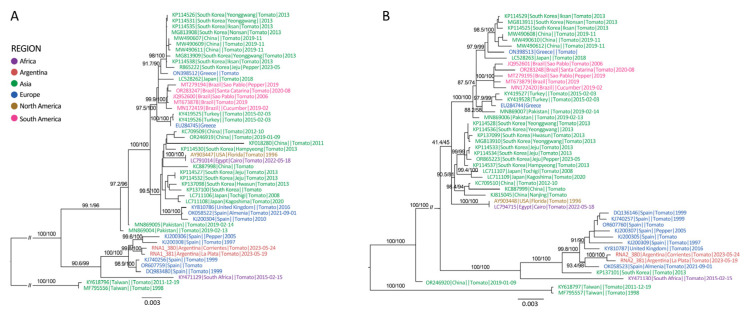
Maximum-likelihood phylogenetic tree of tomato chlorosis virus (ToCV) RNA1 (**A**) and RNA2 (**B**) open reading frame (ORFs) sequences. Statistical support (SH-aLRT/UFBoot) is shown at nodes for some groups. Sequence names (Genbank ID|Country|City|Host|Collection date) are colored by region. The scale indicates the number of substitutions per site.

Phylogenetic analysis revealed a basal diversification event, which separated most sequences into a primary clade, distinct from two divergent sequences from Taiwan (RNA 1 and RNA 2) and one from China (RNA 2) (Figure 4). The primary clade subsequently diverged into two major subclades: one mainly comprised of sequences from Asia, Brazil, and various European countries, including the earliest reported sequence from the USA (AY903447) in 1996, while the other was predominantly formed by sequences from Spain and Argentina, collected between 1997 and 2023 (Figure 4). Notably, sequences from Argentina clustered into a highly supported monophyletic group, reinforcing the hypothesis of a single origin for the virus strains isolated from the Provinces of Buenos Aires and Corrientes (Figure 4).

**Figure 5:**
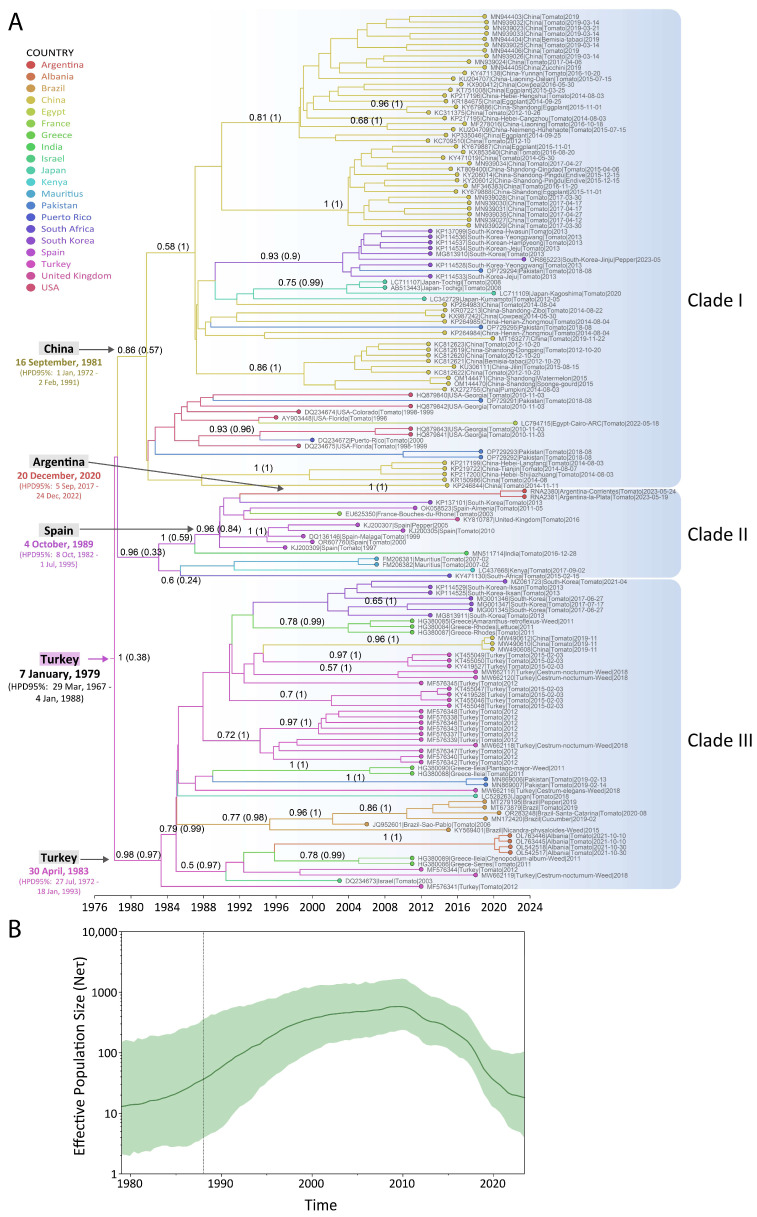
Phylodynamic analysis of ToCV. (**A**) Maximum clade credibility tree for ToCV CP sequences. Posterior values > 0.5 are shown on the branches along with the probability of the ancestral location in brackets. The time scale is in years. (**B**) Demographic reconstruction of ToCV CP sequences. The dark green line shows the median value, and the colored areas represent the 95%HPD interval of the estimated effective population size. The *x*-axis is the time in years, and the *y*-axis is on a log scale (effective population size (Ne) × generation time (Ƭ)).

Phylodynamic analysis of the CP coding region for sequences belonging to the main group of ToCV, excluding the divergent sequences from Taiwan and China, revealed that the ancestor of these sequences began its global diversification in January 1979 (HPD95% = March 1967–January 1988), with Turkey identified as the most probable location (although with low support, ppal = 0.38) (Figure 5A). Clade I originated from an ancestor dating to September 1981 (HPD95% = January 1972–February 1991), with China as the most probable location (ppal = 0.57) (Figure 5A). This clade included sequences from China, South Korea, Pakistan, Japan, Egypt, and the USA. Clade II, which diversified from an ancestor dated to October 1989 (HPD95% = October 1982–July 1995), was most probably located in Spain (ppal = 0.84) and encompassed sequences from Argentina, South Korea, Spain, France, the United Kingdom, India, Mauritius, Kenya, and South Africa, with a basal sequence from India (Figure 5A). The ancestor of the Argentine sequences was dated to late 2020 (HPD95% = September 2017–December 2022), suggesting an introduction from Europe, although a conclusive ancestral location could not be determined (Figure 5A). Furthermore, Clade III began its diversification in April 1983 (HPD95% = July 1972–January 1993), with Turkey identified as the most probable location (ppal = 0.97), and was later spread to South Korea, Greece, China, Turkey, Pakistan, Japan, Brazil, Albania, and Israel (Figure 5A).

Finally, the ToCV global expansion was characterized by an exponential growth in the effective population size from 1979 until 2010, when the main diversification events were taking place, followed by a decline that remains to the present. However, in this last period, the virus entered and diversified into some countries such as Argentina (Figure 5B). Further, the overall substitution rate for the ToCV was estimated as 2.3 × 10^−4^ s/s/y (95%HPD = 1.3 × 10^−4^ − 3.5 × 10^−4^).

### 3.4. Intra-Sample Diversity in Argentine ToBRFV Sequences

**Figure 6:**
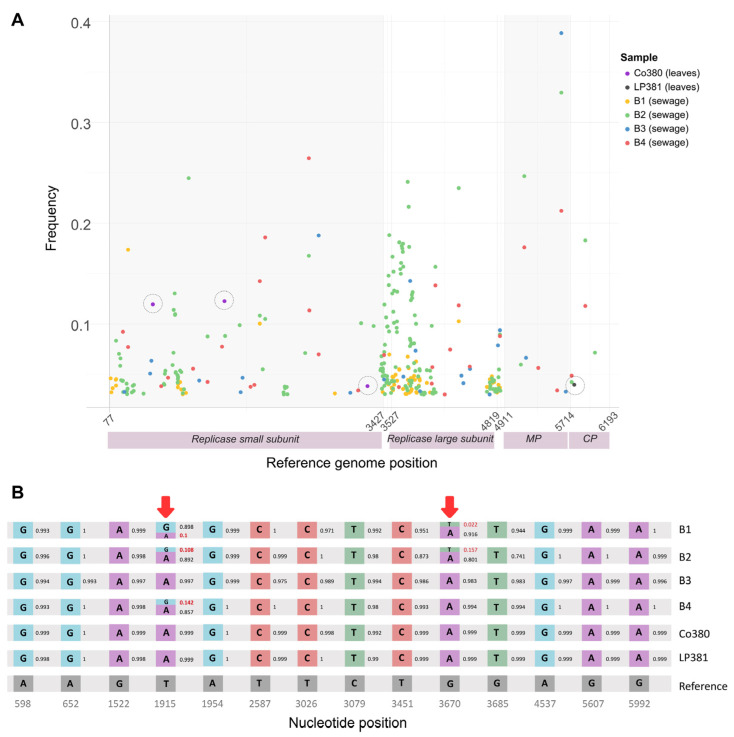
Genomic variation and comparative analysis of the ToBRFV Argentine genomes. (**A**) Distribution of iSNVs in the ToBRFV Argentine sequences. Black dotted circles indicate iSNVs identified in isolates from infected tomato samples. (**B**) Comparative diagram with the NCBI ToBRFV reference sequence. Nucleotide sequence comparison of the coding regions of ToBRFV genomes. The diagram highlights only the positions where iSNVs were identified across all Argentine genomes. Percentages of low-frequency iSNVs (3–50%) are shown in red. Arrows indicate the positions where mixed populations were detected.

To complement the study of ToBRFV sequences from Argentina, the number and frequency of iSNVs were estimated from both plants (*n* = 2) and wastewater samples (*n* = 4) (Figure 6A). For the six Argentine samples, a total number of 312 minor iSNVs (substitutions, insertions, or deletions at frequencies between 0.03 and 0.5) were detected in the ToBRFV coding region (77–6191 nt). The number of low frequencies iSNVs is higher in the sequences obtained from wastewater (Sample 1 = 69, Sample 2 = 186, Sample 3 = 22, and Sample 4 = 31) compared to the ToBRFV isolated from plants (Sample Co380 = 3 and Sample LP381 = 1) (Figure 6A). This indicates that the diversity in environmental samples is much higher than in plant samples. Part of this higher diversity is exemplified by analyzing the signature positions of the Argentine clade, where mixed populations in the wastewater samples can be detected (Figure 6B).

## 4. Discussion

This study highlights the detection of ToBRFV and ToCV in samples collected from different tomato production regions in Argentina, underscoring the significance of these two emerging viruses with global distribution. Furthermore, this research represents the first report of ToCV in Argentina and mixed infections between ToBRFV and ToCV. A previous study documented the mixed infections of ToBRFV and PepMV (cucumber mosaic virus) as well as ToCV and PepMV, although this potexvirus has not yet been reported in Argentina [52,53]. At the same time, co-infections of ToCV and TYLCV (tomato yellow leaf curl virus) exhibited a synergistic effect in tomato plants, leading to more severe symptoms in the later stages compared to single infections and resulting in the breakdown of TYLCV resistance/tolerance [54,55,56,57]. A study conducted in Brazil demonstrated that the latent period was shorter in the co-infection of ToCV and ToSRV (tomato severe rugose virus) than in single infections of these viruses, with plants serving as the inoculum before the onset of symptoms [58]. Although TYLCV and ToSRV have not been reported in Argentina, several begomoviruses infecting tomatoes were reported [59,60]. Within the framework of what has been presented, the occurrence of mixed infections involving both ToBRFV and ToCV raises significant concerns regarding the performance of tomato cultivars’ resistance to ToBRFV. This result highlights the need to develop virus resistance-based management strategies that specifically address mixed virus–virus interactions. In this study, the substitution rate for the ToBRFV genome was estimated as 2.9 × 10^−4^ substitutions per site per year (s/s/y). This rate falls within the range previously estimated for tobamoviruses, which is between 0.1 × 10^−4^ and 10 × 10^−4^ s/s/y [61]. Furthermore, the nucleotide substitution rate for the CP gene of ToCV was estimated at 2.3 × 10^−4^ s/s/y. The mutation rates of both viruses indicate rapid evolution, which may have significant implications for the durability of genetic resistance to infection in crops [62].

The phylodynamic analysis performed in this work identified a single source for ToBRFV outbreaks in the two tomato-producing regions of Argentina, thereby supporting the hypothesis of a unique introduction via contaminated seeds (Figure 3A). The analysis further suggests that the probable ancestral location of the virus could be traced back to Israel.

From a global perspective of the phytogeography results, starting with the North American ToBRFV outbreak, sequences from the USA, Canada, and Mexico were mainly distributed across two distinct clades, Clade H and Clade I. Notably, Clade I contained only sequences from the USA, Mexico, and Canada, whereas Clade H included sequences from these countries as well as from European countries (Netherlands, Belgium, Germany, and the United Kingdom) (Figure 3A). The phylogeographic analysis also revealed that one of the countries with higher viral diversity was the Netherlands, with sequences in Clades A, B, and H (Figure 3A). This wide isolate diversity was also previously shown [63,64,65]. Focusing on South America, Argentina was the second country to report a ToBRFV outbreak, following Peru in 2019. While earlier studies proposed the possibility that ToBRFV originated in South America, the present analyses using the available sequences in public databases and formal evolutionary and phylogeographic models provide stronger evidence supporting Israel as the primary origin, where the virus was first reported [1,2,65,66,67]. These results are consistent with earlier studies [68]. Clade J, which includes sequences from Peru, also contains sequences from various geographical regions, such as Jordan, China, Germany, Italy, the Netherlands, and the USA (Figure 3A). The first reported sequences of ToBRFV, from Israel (KX619418) in 2014 and Jordan (NC028478) in 2015, did not cluster within the same phylogenetic clade (Figure 3A). Notably, the Peruvian sequences share a common ancestor with ToBRFV strains from Jordan, while the Argentine sequences are more closely related to those from Israel (Figure 3A). These findings highlight the following complexities involved in conducting a phylogeographic analysis of a virus with such characteristics: global seed movement, the use of mild ToBRFV strains for cross-protection, and delays in the timely reporting of initial outbreaks. Moreover, these results are based on the information currently available; however, the data are insufficient to establish a conclusive connection regarding some of the events that were involved in the global circulation of ToBRFV. On the other hand, it is difficult to traceback the geographical origin of seed lots because hybrid seeds are often composed of mixed lots of seeds produced in several countries of origin, which increases the probability of association of the virus with the seeds [69]. The International Seed Federation (ISF) has also recognized that the phytosanitary certification of seeds can be challenging because the destination of the seed may not be known when the seed is produced [70]. Previous research highlights the role of infected tomato fruits with ToBRFV on both local and long-distance transmission dynamics, pointing out that management strategies implemented do not consider hygienic measures at growing and packing stations [71].

The phylogenetic analysis of ToCV revealed three clades, as previously shown [72]. The analysis also identified a single source for the sequences from the tomato-producing regions of Argentina (Figure 4). Hypothesizing the plant source of this virus’s introduction in Argentina is challenging due to its lack of seed-borne transmission. As ToCV is transmitted by whiteflies, we anticipated that the Argentine sequences would cluster within the same clade as those from Brazil, reflecting the likely migration of viruliferous whiteflies from Brazil into Argentina, thereby facilitating the transmission of ToCV. The phylodynamic analysis revealed that the Argentine sequences grouped within Clade II, alongside sequences from South Korea and Spain, while the Brazilian sequences were placed in Clade III, sharing this clade with sequences from South Korea, China, Greece, Turkey, Pakistan, Japan, Israel, and Albania, thus refuting the hypothesis of a direct dispersion from Brazil to Argentina by whiteflies (Figure 5A). ToCV has been detected in Brazil since 2006 and in Uruguay since 2012; however, no sequences have been reported from the latter country to date [73,74]. The first reported sequence (AY903448) from the USA in 1996 clustered within Clade I, where it is related to other sequences from the USA and more distantly—but sharing the clade—with Chinese and other Asian sequences (Figure 5A). Although the first reported case of ToCV was in the USA, the most likely ancestral origin, based on the current database, is Turkey. Given that the tree clade includes sequences from countries located on different continents, and that three distinct introduction events were identified in the Americas (USA, Argentina, and Brazil), we raise the question of what role the movement of seedlings across borders and the virus’s broad host range play in the spread of ToCV. This underscores the need for reflection on the measures that should be implemented worldwide to prevent future viral outbreaks that could severely impact tomato production.

The phylodynamic analysis revealed different times for the ancestral diversification, predicting that ToCV (December 2020) began its circulation before ToBRFV (August 2021) in Argentina (Figure 5A and Figure 3A). The outbreak of ToBRFV and ToCV in Argentina occurred during the decline phases of the global spreading of both viruses, as observed in the effective population size curves estimated in this work (Figure 3B and Figure 5B). For ToBRFV, the reduction in population size post-2020 coincides with the onset of the COVID-19 pandemic, likely reflecting a decrease in seed movement. This decline may also be attributed to the substantial efforts made to implement phytosanitary measures aimed at curbing the global spread of ToBRFV.

In addition to detecting ToBRFV in plants, the virus was also identified in wastewater in Argentina in samples collected several months before symptomatic infections were reported in plants. In Argentina, wastewater samples were collected in Buenos Aires, starting in February. The first report of ToBRFV in tomatoes cultivated in the Province of Corrientes was documented in December 2022 [3]. These two locations, situated around 800 km apart in Argentina, underscore the widespread distribution of ToBRFV, and this emphasizes the utility of the high-throughput sequencing analysis of wastewater metagenomes for the early detection of emerging and quarantine plant viruses within agricultural regions. This work also demonstrates that the population diversity of ToBRFV in wastewater is greater than in individual plants (Figure 6), highlighting the importance of this tool to complement the monitoring of symptomatic plants, helping both to obtain consensus genomes for evolutionary analysis that could affect the effectiveness of management strategies based on virus resistance.

The Argentine outbreak exemplifies the profound consequences of the international movement of contaminated seeds, fruits, and plant materials, highlighting the critical role this movement plays in the global dissemination of viruses. The primary determinants of disease emergence include host susceptibility, environmental conditions, and pathogen virulence, as conceptualized in the “disease triangle” [75,76]. Additionally, the health triangle framework, which addresses the diagnosis, prediction, restoration, and maintenance of host health, offers significant advantages, particularly when considering the role of the microbiota [77]. However, a key limitation of this framework is its human activity, a fundamental factor influencing the onset of diseases and health disturbances in cultivated plants. Agricultural practices, land-use changes, and global trade significantly shape plant health, both directly and indirectly, by modifying environmental conditions and pathogen dynamics. Therefore, incorporating the influence of human activity into this framework is essential for a more holistic understanding of plant health and the development of effective disease management strategies.

## Data Availability

The data presented in this study are available in GenBank under accession numbers PV146433, PV146434, PV289029, PV289030, PV289031, PV289032, PV300244, PV300245, PV300246, and PV300247.
